# Lifting the innate immune barriers to antitumor immunity

**DOI:** 10.1136/jitc-2020-000695

**Published:** 2020-04-08

**Authors:** Carla V Rothlin, Sourav Ghosh

**Affiliations:** 1 Immunobiology, Yale School of Medicine, New Haven, CT 06519, United States; 2 Pharmacology, Yale School of Medicine, New Haven, CT 06519, United States; 3 Neurology, Yale School of Medicine, New Haven, CT 06519, United States

**Keywords:** immunity, innate, dendritic cells, inflammation, lymphocyte activation, macrophages

## Abstract

The immune system evolved for adequate surveillance and killing of pathogens while minimizing host damage, such as due to chronic or exaggerated inflammation and autoimmunity. This is achieved by negative regulators and checkpoints that limit the magnitude and time course of the immune response. Tumor cells often escape immune surveillance and killing. Therefore, disrupting the brakes built into the immune system should effectively boost the anticancer immune response. The success of anti-CTLA4, anti-PD-1 and anti-PD-L1 have firmly established this proof of concept. Since the response rate of anti-CTLA4, anti-PD-1 and anti-PD-L1 is still limited, there is an intense effort for the identification of new targets and development of approaches that can expand the benefits of immunotherapy to a larger patient pool. Additional T cell checkpoints are obvious targets; however, here we focus on the unusual suspects—cells that function to initiate and guide T cell activity. Innate immunity is both an obligate prerequisite for the initiation of adaptive immune responses and a requirement for the recruitment of activated T cells to the site of action. We discuss some of the molecules present in innate immune cells, including natural killer cells, dendritic cells, macrophages, myeloid-derived suppressor cells, endothelial cells and stromal cells, that can activate or enhance innate immune cell functions, and more importantly, the inhibitors or checkpoints present in these cells that restrain their functions. Boosting innate immunity, either by enhancing activator functions or, preferably, by blocking the inhibitors, may represent a new anticancer treatment modality or at least function as adjuvants to T cell checkpoint inhibitors.

## Beyond current immune checkpoints

Immune checkpoint inhibitor therapy (ICT), such as with anti-CTLA4 (ipilimumab), anti-PD1 (nivolumab, pembrolizumab and cemiplimab) or anti-PD-L1 (atezolizumab, avelumab and duravalumab), has resulted in significant and durable clinical response in a subset of patients with certain types of cancer.^1 2^ ICT is U.S. Food and Drug Administration (FDA) approved for 16 indications including unresectable or metastatic melanoma, advanced non-small cell lung cancer (NSCLC), advanced small cell lung cancer, advanced head and neck squamous cell carcinoma, classical Hodgkin lymphoma, refractory primary mediastinal large B-cell lymphoma, certain advanced urothelial carcinomas, certain gastric cancers, advanced oesophageal cancer, advanced cervical cancer, hepatocellular carcinoma (HCC) previously treated with sorafenib, advanced Merkel cell carcinoma, advanced renal cell carcinoma, certain endometrial cancers, microsatellite instability high (MSI-H) or deficient mismatch repair (dMMR) metastatic colorectal cancer, and MSI-H cancers. Although that the ICT market is predicted to be worth US$7 billion by 2020,^1^ the estimated number of responders to ICT in 2018 was only a modest 12.46%.^1^ The majority (~57%) of patients with cancer still do not qualify for ICT.^1^ Many cancers remain intractable to immune checkpoint inhibitors. Breast, non-MSI-H and non-dMMR colorectal or prostate cancer, for example, are mostly unresponsive to ICT therapy. Even among the cancers that respond to ICT, only a subset of patients benefits from this treatment. In NSCLC, a cancer with the highest response estimate, one study determined a successful response in only 7.09% patients,^1^ although other studies indicate investigator-assessed objective response rate of 41% in treatment-naïve patients and 23% in previously treated patients.^3^ For ICT to be truly the watershed in cancer treatment, this modality needs to be extended to and be effective in a significantly larger group of cancer patients.

The success of current ICT is based on a fundamental understanding of the principle that molecular signals constrain active T cells during their effector functions.^4 5^ Current efforts towards improving the efficacy and the reach of ICT can be broadly grouped into a couple of approaches. One approach is essentially iterative: targeting additional T cell checkpoint inhibitors such as T cell immunoglobulin domain and mucin domain-3 protein (TIM-3), lymphocyte activation gene-3 protein, T cell immunoreceptor with Ig and ITIM domains (TIGIT) or costimulatory molecules such as 4-1BB or OX40 and their ligands.^6–9^ Yet, the principle that can predict the engagement of a specific checkpoint over others in a cancer or a molecular subtype of cancer remains undiscovered. At this time, the approach of identifying the specific T cell checkpoints to be targeted when anti-PD-1/anti-PD-L1 or anti-CTLA4 fail is primarily based on trial and error. A different approach is combination therapy or including additional therapeutic modalities to anti-PD-1/anti-PD-L1 or anti-CTLA4. For example, based on the findings that high versus low mutation burden of the tumor improves ICT,^10–14^ the combination of ICT with other conventional treatment options, such as chemotherapy or radiotherapy, has been proposed as a more effective way to enhance antitumor immunity.^15^

Is there a way to rationally identify and reverse some of the molecular or cellular factors limiting the efficacy of current ICT? One important correlate of success versus failure of anti-CTLA4 is the presence of a molecular signature of a pre-existing T cell response in the tumor tissue or so-called T-cell inflamed tumors.^16 17^ Other factors that positively correlate with the efficacy of ICT include favorable gut microbiome,^18 19^ Batf3^+^ dendritic cells (DCs),^20^ activation of the stimulator of interferon genes (STING) pathway,^21^ type I interferons (IFNs),^22^ signatures of wound healing and obesity,^23 24^ while negative correlates include unfavorable gut microbiome,^25^ Wnt/β-catenin signaling^17^ and stromal factors in the tumor microenvironment such as TGFβ.^26^ Implicit in the categorization of the molecular correlates of ICT is the understanding that signals that are not intrinsic to T cells can determine the success or failure of ICT. We posit that identifying and targeting immune regulators beyond T cells can drive a new frontier in cancer immunotherapy. Here, we primarily focus on a distinct premise—the innate immune response and the tumor microenvironment as critical determinants of the antitumor response.

**Figure 1:**
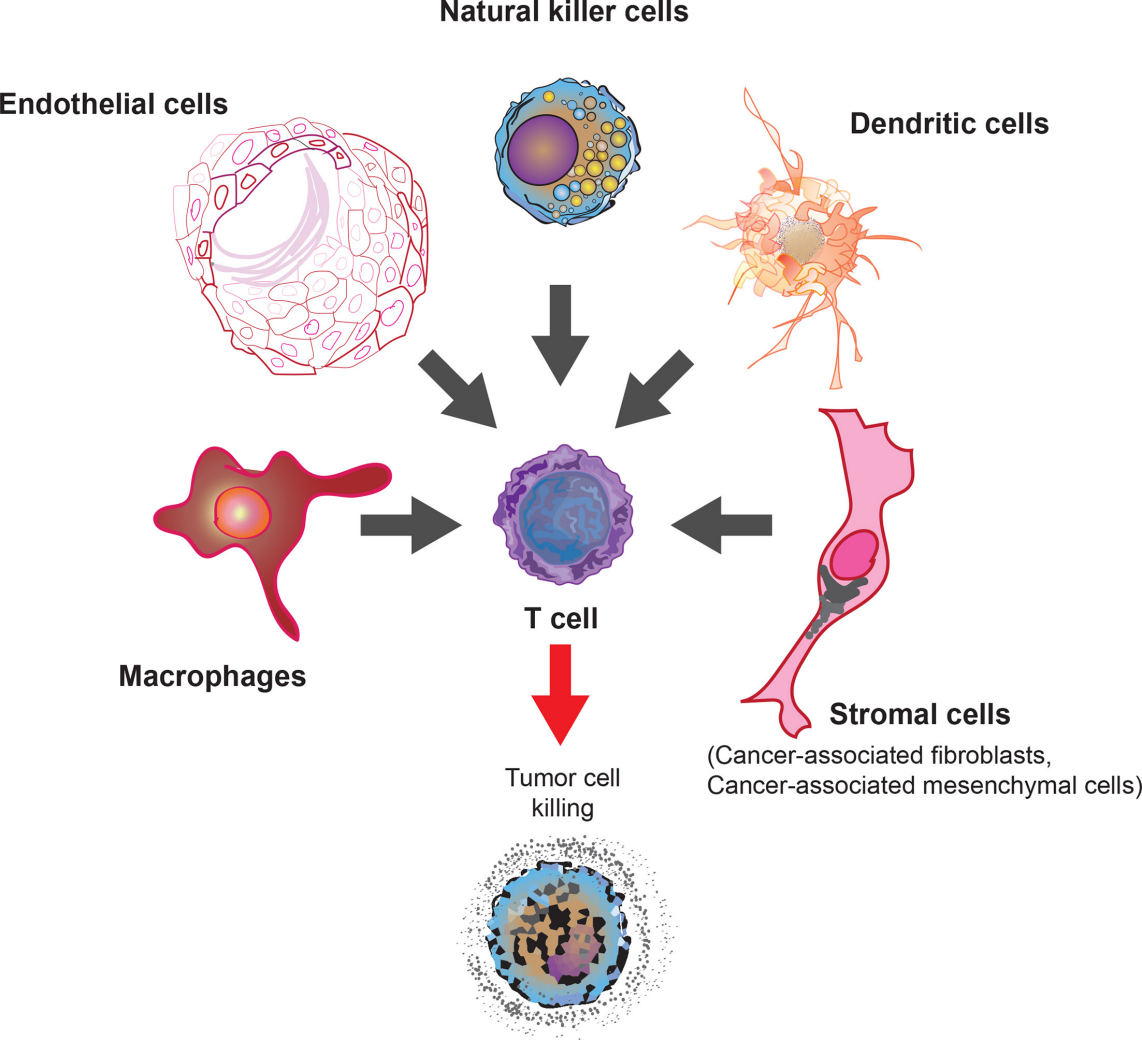
A constellation of innate immune cells sculpts the environment for effective T cell-mediated killing of cancer cells. While T cells are commonly the effectors of antitumor immunity, a panoply of innate immune cells including macrophages, dendritic cells, natural killer cells, endothelial cells and stromal cells such as cancer-associated fibroblasts and mesenchymal cells can regulate the efficiency of T cell activation and tumor infiltration.

**Figure 2:**
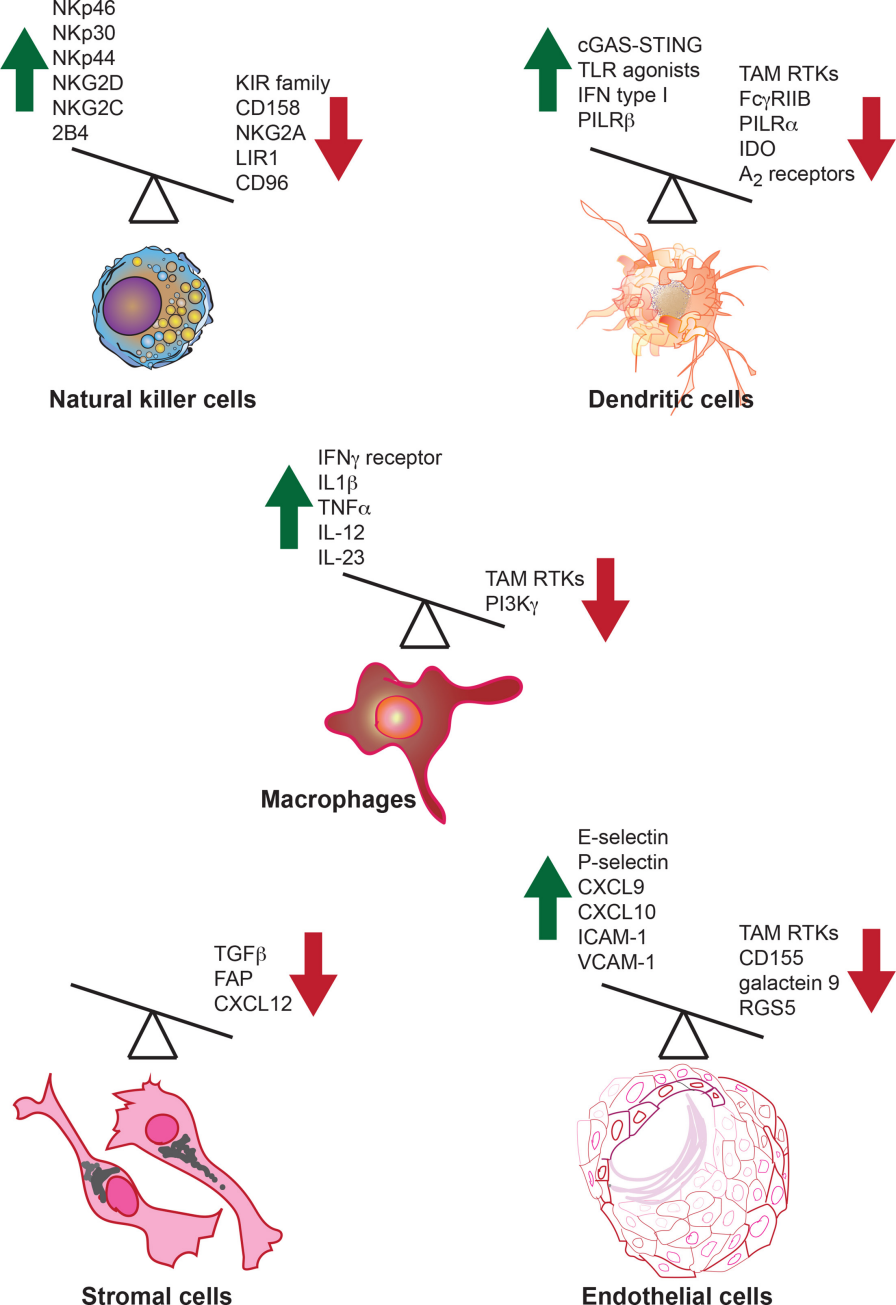
Activating and inhibitory molecules in innate immune cells regulate their antitumor immune functions. Both activating and inhibitory molecules regulate innate immune cell function or are produced by innate immune cells in the context of the antitumor immune response. A few examples of activators and inhibitory molecules that regulate or are produced by innate immune cells are shown. Green upward arrow indicates molecules that enhance antitumor immunity, while red downward arrow indicates molecules that inhibit the antitumor immune response. Overexpression or induction of the function of activators can boost antitumor immunity; similarly, the blockade of the inhibitors that dampen antitumor immunity should also drive a more effective antitumor response. FAP, fibroblast activation protein; IDO, indoleamine 2,3-dioxygenase; IFN, interferon; IL, interleukin; RTKs, receptor tyrosine kinases; TLR, Toll-like receptor; TNFα, tumor necrosis factor α.

The innate immune system constitutes the first line of host defense. Innate immunity consists of physical and chemical barriers to infection, as well as different cell types dedicated to the broad-spectrum pattern-based recognition of microorganisms. One of the earliest steps during an immune response, for antiviral defense, for example, involves the stimulation of innate immune cells such as natural killer (NK) cells and DCs. A fundamental principle or characteristic of the immune response is that the engagement of the innate immune system is an obligate prerequisite for induction of T cell responses.^27^ Adaptive immunity, which follows the innate immune response, eliminates invading pathogens through specific recognition of the identity of the microbe (microbial antigens) and establishes immunological memory. However, antigen-specific functions of T cells cannot be engaged without innate immune cells. Even once T cells are activated, the innate immune response can affect their effector functions (figure 1). Furthermore, not only are T cell responses dependent on the activation of innate immune cells, there are also effective ways of immune-mediated killing of tumor cells that are independent of T cells, such as by NK cells—a cell that is part of the innate immune system. Importantly, the innate immune cells are themselves regulated by built-in activators and inhibitors. Here, we describe cells and molecules that function in innate immunity, primarily by regulating the magnitude, quality and period of the immune response. Innate immune cells and the molecules that regulate their function represent attractive candidates for improving the antitumor immune response—or at least complementary approaches to T cell immune checkpoint inhibitors for therapeutic targeting. Below, we discuss critical molecules and pathways (activators and inhibitors) that can regulate the magnitude of the antitumor immune response (figure 2), and might represent rational non-T cell immune checkpoints.

## Boosting and/or harnessing the activators of innate immunity for improving antitumor responses

If we know the activators of innate immunity, we can add more of it for improving innate immune function for a more efficient antitumor response. NK cells belong to the family of innate lymphoid cells and are highly efficient in detecting and destroying virally infected cells or tumor cells.^28^ NK cell activity is positively regulated by signals from activating receptors such as NKp46, NKp30, NKp44, NKG2D, NKG2C, 2B4.^29^ Additionally, NK cell cytotoxic activity is promoted by the cytokine interleukin (IL)-15 that signals through components of the IL-2 receptor (IL-2R).^30^ Expanding the number of NK cells, such as through PEGylated IL-2, either as monotherapy or in combination with ICT (Nektar Therapeutics) or IL-15 superagonist/IL-15 receptor α fusion complex (ALT-803), is being explored.^31^ A table listing the active clinical trials for IL-2, IL-15 and other targets discussed below can be found in supplementary materials (online supplementary table).

10.1136/jitc-2020-000695.supp1 Supplementary data

10.1136/jitc-2020-000695.supp2 Supplementary data

Pharmacological approaches targeting NK cell activating receptors are being investigated as anticancer therapies. NK cells expressing a chimeric-antigen receptor (CAR) NKG2D-DAP10-CD3ζ were shown to be highly cytotoxic against leukemia and solid tumor lines.^32^ NK cells have been engineered to express anti-B-cell antigen CD19 single-chain fragment (anti-CD19scFv CAR) as well as a fusion receptor consisting of IL-15 with IL-15 receptor α for autonomous IL 15 stimulation that increases the lifespan of NK CARs after infusion.^33^ Anti-CD19 CAR NK cells are in clinical trials in relapsed and refractory B cell lymphoma (Alllife; clinicaltrials.gov identifier: NCT03690310). A proteolytic cleavage-resistant CD16 Fc receptor is also added to some CAR NK cells (Fate therapeutics). Other NK CARs for B-cell acute lymphoblastic leukemia includes Fms-related tyrosine kinase 3 (FLT3)-specific NK CARs containing CD28-CD3ζ along with icasp9 (inducible caspase nine suicide gene)^34^ or CD22-CAR engineered to secrete CD-19 engager.^35^

Another recent strategy for NK cell activation is based on bifunctional and trifunctional killer cell engagers (BiKEs and TriKEs) that are designed to trigger antigen-specific NK cell-mediated tumor killing. For example, bispecific antibodies have been designed to target tumor-specific antigens and the NKp46 activating receptor on NK cells (Innate Pharma).^36^ The Fc portion of tumor-specific antibodies bind CD16, a type III Fcγ receptor on NK cells that can activate antibody-dependent cell-mediated cytotoxicity against tumor cells. Therefore, CD16-directed bispecific (eg, CD16 and CD19) or trispecific (eg, CD16 and two tumor antigens CD19 and CD22 or one tumor antigen and an IL-15 linker) antibodies or scFv have been developed.^37^

Although primarily functioning as innate immune cells, studies have shown that NK cells might also have a role in antigen-specific immunological memory. In a Rag2-deficient model of hapten-induced contact hypersensitivity, NK cell-mediated hapten-specific response was detected for at least a month after priming.^38^ Similar NK cell memory was demonstrated against mouse cytomegalovirus (MCMV).^39–41^ Whether similar long-lasting antitumor NK response exists remains unknown. NK cell-mediated tumor killing may also impact downstream T cell responses. NK cells were shown to accelerate CD8 T cell responses against MCMV by negatively regulating immunosuppressive plasmacytoid DC cytokine production.^42^ Chemokines and cytokines produced by NK cells, such as CCL5, can recruit conventional DCs and prime their antitumor function.^43^ Such NK cell-mediated accumulation of a DC subtype was demonstrated in BRAF^V600E^ mouse model of melanoma.^43^ Another study demonstrated that NK-derived FLT3 ligand (FLT3L) can activate intratumoral DCs and NK cells predict anti-PD-1 responsiveness in patients with melanoma.^44^ NK cell-mediated killing of target cells lacking MHC class I was also demonstrated to induce a robust CD4^+^ and CD8^+^ T cell response against cells.^45^ The antigen-agnostic ability of NK cells to target abnormal or altered somatic cells and the absence of need to undergo effector differentiation makes them attractive candidates as immunotherapy agents. However, as discussed next, NK cells activity can be dampened by secreted products from tumor cells such as prostaglandin E2 (PGE2).^43^ Proteolytic shedding of NKG2D ligands such as MHC class I-related chain A (MICA) and B (MICB) by tumors can also enable their escape from NK cell-mediated killing by functioning as decoys and driving NK cell exhaustion.^46 47^

Other innate immune cells are also being harnessed for immunotherapy such as DCs. DCs can take up foreign antigen and cross present these to T cells to trigger cytotoxic responses. Cross-presenting DCs have been shown to be required for mounting an effective immune response against immunogenic tumors in mice.^48 49^ The process of cross presentation is facilitated by specific cytokines. For example, type I IFNs are markedly upregulated in response to viral infections and promote the ability of DC cross priming and activation of cytotoxic T cells.^50^ In principle, this strategy is equally applicable to cancer antigens. Indeed, type I IFNs have been shown to be required for mounting an effective antitumor response. Genetic ablation of type I IFN receptors or neutralization of their function rendered mice more susceptible to tumor growth.^51 52^ Consistent with these findings, Roferon-A (IFN α−2a; Roche) is approved for the treatment of patients with hairy cell leukemia and AIDS-related Kaposi’s sarcoma, while Intron A (IFN α−2b; Merck) is approved for hairy cell leukemia, malignant melanoma, follicular lymphoma and AIDS-related Kaposi’s sarcoma. DC-based efforts are, however, susceptible to adaptive changes in the tumor cell. Constant pressure from the immune system together with the genetic instability of tumor cells can lead to the selection of immunoedited tumor subclones that have lost the neoantigens or masked those. For example, analyses of matched tumor tissue from pretreatment patients with NSCLC and postemergence of acquired resistance to ICT revealed loss of 7–18 putative mutation-associated neoantigens.^53^

Type I IFNs are expressed in response to the activation of specific pattern-recognition receptors, such as Toll-like receptor (TLR) 7 and TLR 9. This has led to the development of pharmacological approaches to activate these receptors intratumorally. Multiple TLR 9 agonists, including SD-101 (Dynavax), CMP-001 (Checkmate Pharmaceuticals) and IMO-2125 (Idera Pharmaceuticals) are being tested in Phase 1 and 2 clinical trials in lymphomas or various solid tumors in combination with standard of care or immune checkpoint inhibitors (clinicaltrials.gov identifiers: NCT02521870, NCT03007732, NCT03831295, NCT03410901, NCT03322384, NCT02254772, NCT01042379, NCT02680184, NCT03084640, NCT03618641, NCT03507699, NCT03438318, NCT03445533, NCT02644967, NCT03865082, NCT03052205). Similarly, agonists of TLR3 such as Poly-ICLC or Hiltonol (Oncovir) and BO-112 (Bioncotech Therapeutics) are in clinical testing in several cancers including prostrate, breast, colon, head and neck cancers, sarcomas, glioblastoma and low-grade gliomas (including clinicaltrials.gov identifiers: NCT02423863, NCT03665545, NCT03262103, NCT02643303, NCT02834052, NCT02828098). TLR7/8 agonists such as NKTR-262 (Nektar Therapeutics), CV8102 (CureVac AG) and LHC-165 (Novartis) are also being tested (clinicaltrials.gov identifiers: NCT03435640, NCT03203005, NCT03291002, NCT03301896).

Another approach for boosting antitumor immunity is centered on the recently identified intracellular DNA-sensing cGAS-STING pathway. The cGAS-STING pathway is important for the induction of type I IFNs and effective antitumor immunity.^21^ Intratumoral or intraperitoneal delivery of a murine STING agonist DMXAA resulted in tumor regression.^54^ Based on this success, intratumoral administration of the human STING agonist (MK-1454, Merck) entered clinical trials (clinicaltrials.gov identifier: NCT 03010176). Unfortunately, this approach was not effective as monotherapy, although it showed partial response in combination with anti-PD-1 in patients with advanced solid tumors or lymphomas.^55^ Another STING agonist (ADU-S100, Aduro Biotech), in combination with anti-PD1 or anti-CTLA4, is in multiple clinical trials including head and neck, advanced/metastatic solid tumors or lymphomas (clinicaltrials.gov identifier: NCT03937141, 02675439, 03172936). A recent presentation on the first outcomes of this clinical trial revealed lower response rates than those expected (treatment discontinued in 74% of patients), but partial response was observed in patients with PD-1 naïve triple negative breast cancer and PD-1 relapsed/refractory melanoma.^56^

The type II IFN, interferon gamma (IFNγ) has antiviral, immunoregulatory, and antitumor properties. While IFNγ signaling in tumor cells leads to upregulation of PD-L1, transporter associated with antigen processing (TAP1), MHC class I and T cell chemotaxis factors, as well as IFN-induced growth inhibition, IFNγ is also a key regulator of DCs and macrophages. IFNγ polarizes macrophages towards a proinflammatory phenotype characterized by elevated production of IL1β, IL-12, IL-23, tumor necrosis factor α (TNFα) and nitric oxide (NO).^57^ IFNγ also enables cross presentation.^58^ Acquired resistance to PD-1 (pembrolizumab) correlated with somatic loss-of-function mutations in *JAK2*, a gene encoding a tyrosine kinase required for IFNγ signaling, in patient tumors.^59^ While the loss of IFNγ signaling directly affected IFN-induced growth inhibition of tumor cells,^59^ similar germline mutations might also correlate with reduced ICT via reduced cytokine production by tumor-associated macrophages or as a consequence of reduced cross presentation.

The induction of type I or type II IFN notwithstanding, an appropriate population of intratumoral DCs is required for mounting an antitumor immune response. Are DCs present in sufficient numbers in the tumor? The abundance of intratumoral DCs was found to be dependent on the production of the cytokine FLT3L produced in part by NK cells.^44^ The pharmacological administration of FLT3L together with the TLR 3 agonist Poly I:C expanded DC numbers and enhanced the response to targeted and immunotherapy in mouse models.^60^ Even if DCs are present in sufficient numbers, is their capacity to migrate to the draining lymph node important vis-à-vis the induction of an effective antitumor response? CCR7 was found to be required for DC migration and T cell priming in the tumor draining lymph node of mice.^61^ Of note, the expression of *CCR7* in human tumor samples, highly correlated with the expression of *CD3E*, suggests that the same chemokine receptor is relevant in humans.^61^

## Blockade of negative regulators or checkpoints of innate immunity for improving antitumor responses

*In ‘Through the Looking Glass’ Alice ran as fast as she could only to observe: ‘Why, I do believe we’ve been under this tree the whole time! Everything’s just as it was!’*. Through the Looking-Glass, Lewis Carroll, 1871.

Homeostasis mandates that pathways to activate a system are balanced by opposing pathways that can ensure return to the baseline. Hence, there are a number of molecules that function as negative regulators or checkpoints during immune activation. For example, immunoreceptor tyrosine-based activation motifs or ITAMs are often inexorably functionally linked to immunoreceptor tyrosine-based inhibitory motifs or ITIMs within an immune cell. Sometimes, the faster you accelerate, the harder the brakes come on to prevent exaggerated responses. Therefore, to drive an effective antitumor response, it is often not just sufficient to push the accelerator (activating mechanisms) but to additionally disengage the brakes (inhibitory mechanisms). As described above, ITIM-containing inhibitory receptors such as PD-1 and TIGIT have been targeted to boost antitumor immunity. The immune system—both innate and adaptive—is replete with examples of activation mechanisms hardwired with autologous inhibitory circuit breakers to limit the magnitude of the response. Here, we focus on examples from innate immune cells. As an interesting aside, PD-1 and TIGIT are not only expressed in T cells but also in NK cells^29^ and an NK cell-dependent effect was indispensable for the full therapeutic benefit of PD-1/PD-L1 blockade in several mouse models of cancer.^62^ ITIMs are found not only in PD-1 and TIGIT but also in receptors in NK cells that mediate the recognition of HLA-A, B and C molecules and tolerance to self, for example, the KIR family members KIR2DL1, KIR2DL2, KIR2DL3, KIR2DL5, KIR3DL1, KIR3DL2, and KIR3DL3.^29^ When NK cell receptors recognize the MHCI expression on target cells as self, NK cells are ‘turned off’ from killing. Infected cells, as well as tumor cells, often lose MHCI molecules. The lack of MHCI is a green light for NK cells to unleash their killing capacity. Other inhibitory receptors include CD158, NKG2A and LIR1.^29^ Blockade of inhibitory receptors can enhance NK cell tumoricidal activity. Monalizumab (Innate Pharma) is an NKG2A-blocking antibody in clinical trials, including in combination with cetuximab (EGFR inhibitor) or durvalumab.^63^ Monalizumab trials are underway in hematological malignancies and in solid tumors such as renal cancer (see online supplementary table or clinicaltrials.gov for identifiers). It is to be noted that NKG2A is also expressed by a subset of intratumoral CD8 T cells after cancer vaccine treatment. In one study, inhibition of this receptor or its ligand potentiated cancer vaccine responses in a CD8 T-dependent, NK cell-independent manner.^64^

What are some other inhibitory receptors on innate immune cells that can be potential targets for ICT? ITIM containing receptors play an important role in DC maturation. DCs derived from mice deficient for the ITIM containing FcγRIIB can generate improved antigen-specific T cell responses in vitro and in vivo.^65^ Genetically ablating these receptors or blocking these receptors allows DCs to mature spontaneously and upregulate CD80, CD86 and MHC II molecules,^66 67^ suggesting that these receptors can be targets for improving cancer immunotherapy. FcγRIIB receptors were demonstrated to promote the internalization of rituximab and thereby inhibit macrophage-dependent phagocytosis of malignant B cells in chronic lymphocytic leukemia (CLL) and mantle cell leukemia.^68^ An antagonistic human FcγRIIB antibody was found to be effective in mice xenografts of primary, as well as CD20-refractory CLL.^69^ This effect was mediated, in part by preventing the internalization of rituximab from the surface of malignant cells, and in part by direct cytotoxicity.^69^

Another example of an ITIM-containing receptor is SIRPα. The ligand for SIRPα is CD47, a ‘do not eat me’ signal that was found to be increased on stem cells of patients with acute myeloid leukemia (AML).^70 71^ The observation that CD47 is highly expressed on tumor cells was expanded to most of cancers including ovarian, breast, colon, bladder, prostate, HCCs and glioblastoma.^72^ Blockade of CD47–SIRPα interaction leads to the activation of innate immune cells such as macrophages and neutrophils and cancer cell killing by calreticulin-activated phagocytosis.^73^ CC-95251 (Celgene), an anti-SIRPα antibody, is in clinical trials in patients with advanced solid or hematological cancers (clinicaltrials.gov identifier: NCT03783403). Hu5F9-G4, a humanized monoclonal antibody against CD47, is in trials as a monotherapy or in combination with azacitidine in hematological malignancies (clinicaltrials.gov identifier: NCT03248479) or in combination with rituximab in relapsed/refractory B-cell lymphoma and solid tumors including advanced colorectal cancer (clinicaltrials.gov identifiers: NCT02953509, NCT02953782). Hu5F9-G4 is also under testing in combination with anti-PD-L1 (clinicaltrials.gov identifier: NCT03922477). A CD47 blocking antibody, TTI-621 (Trillium), is also in clinical trials for hematological malignancies and selected solid tumors (clinicaltrials.gov identifier: NCT02663518). It is to be noted however that the blockade of CD47–SIRPα interaction with an intact antibody or other tumor opsonizing antibodies also drives antibody-dependent cellular phagocytosis or cytotoxicity by FcRγ-expressing cells such as macrophages, neutrophils or NK cells. More recently an alternative "do not eat me" pathway driven by another ITIM containing receptor—SIGLEC-10—was identified. Specifically, SIGLEC-10 was found to be highly expressed by tumor-associated macrophages and mediated an antiphagocytic signal in the context of several tumors expressing its ligand CD24.^74^ Determining the specific engagement of CD47-dependent and CD24-dependent pathways in different tumor types will be relevant for the development of personalized immunotherapies based on the blockade of "do not eat me" signals.

Extending this paradigm, any receptor with an ITIM motif and with known functional role in antagonizing activating signals may be a potential ICT target. CD226 is a costimulatory adhesion molecule expressed by T cells and NK cells.^75–77^ CD155, expressed on transformed cells,^75 76^ can engage CD226. CD96 and TIGIT are ITIM-containing receptors that function as inhibitors of CD226 by competing for CD155 binding.^75–77^ *Cd96* ^−/−^ NK cells produce increased amounts of IFNγ and blocking CD96 inhibited metastasis in mouse models of B16F10 and LWT1 melanoma, 3LL lung cancer and RM-1 prostate carcinoma.^78 79^ Furthermore, blockade of CD96 in *Tigit* ^−/−^ mice improved the reduction of B16F10 melanoma or EO771 lung metastasis.^79^ CLEC12B also contains an ITIM motif and can antagonize NKG2D-mediated signaling, although its cellular and molecular function is not well characterized.^80^ Neutrophils, monocytes, macrophages and DCs also express the activating PILRβ (activating FDF03) and inhibitory PILRα (inhibitory FDF03).^81^ PILRα contains ITIMs while PILRβ associates with the ITAM-containing adapter DAP12.^82^ *Pilrb* ^−/−^ macrophages produced lower amounts of TNFα and IL-1β and higher amounts of IFN-γ and IL-12p70 when challenged with *Staphylococcus aureus*.^83^ The possible utility of targeting the PILRα/β-receptors in cancer is exemplified by a patent (US8178094B2).

Another receptor that can mediate exhaustion in NK cells is TIM-3. Although TIM-3 does not contain ITIM motifs, its expression was upregulated in NK cells in patients with melanoma and correlated with poor prognosis.^84^ NK cells purified from peripheral blood displayed an exhausted phenotype, and this exhaustion was reversed by soluble TIM-3 blocking antibodies.^84^ TIM-3 engagement can block NKG2D-induced killing, much like CD94.^85^ Currently, anti-TIM-3 antibody TSR-022 is being tested in a multicenter, open-label phase 1 study as monotherapy and in combination with anti-PD-1 in patients with advanced solid tumors (Tesaro, clinicaltrials.gov identifier: NCT02817633). Another anti-TIM-3 agent (MGB453; Novartis), in combination therapy, is in trials including in recurrent glioblastoma (clinicaltrials.gov identifiers: NCT03961971, NCT02608268, NCT03946670). A different anti-TIM-3 antibody (LY3321367; Eli Lilly) is also being tested in another trial (clinicaltrials.gov identifier: NCT03099109).

Specific protein–protein interaction motifs can also function to disrupt aggregative activating signaling and function as checkpoints for inflammatory signaling. IL-1R8/SIGIRR counteracts TLR and interleukin 1 receptors (IL1R)-dependent activation.^86^ IL-1R8 contains TIR domains. These domains are found in receptors such as TLRs, which dimerize on ligand binding. TIR domains are also found in critical cytoplasmic adaptors such as MyD88 and Mal/TIRAP, which are recruited to the receptors such as TLRs via TIR–TIR interactions thus driving downstream signaling cascades. Unlike the TIR domains described above, the IL-1R8 TIR domain interferes with the TIR-domain associations of receptors during formation of the Myddosome and functions as a negative regulator of inflammatory signaling.^87^ IL-1R8 also functions as the coreceptor of IL-1R5/IL-18Rα for the anti-inflammatory cytokine IL-37.^88^ *Il1r8* ^−/−^ mice are characterized by increased frequency and absolute numbers of NK cells and earlier NK cell maturation (by 2–3 weeks of age).^89^ Expression of NKG2D, DNAM-1 and Ly49H NK cell activating receptors were enhanced and the cells were more proficient in IFNγ, Granzyme B and FasL production. In a model of diethylnitrosamine-induced HCC, *Il1r8* ^−/−^ mice demonstrated improved protection, which correlated with increased production of IFNγ and reduced levels of inflammatory cytokines and chemokines such as IL-6, TNFα, IL-1β, CCL2 and CXCL1.^89^ Similarly, *Il1r8* ^−/−^ mice showed reduced lung metastasis in a MN/MCA1 sarcoma model and liver metastasis in MC38 colon cancer model. Furthermore, the protection against HCC and lung metastasis was mediated by NK cells.^89^ IL-1R8 is also upregulated in breast cancers.^90^

Ectoenzymes can also profoundly negatively regulate the immune response, including innate immunity. Extracellular ATP (eATP) is serially converted into ADP, AMP and adenosine by a group of ectoenzymes that include CD39 and CD73.^91^ eATP can signal through a panoply of purinergic receptors that induce the activation and migration of myeloid cells.^92 93^ Of note, eATP can also also affect adaptive immunity, by enhancing effector T cell function while inhibiting the differentiation of immunosuppressive Tr1 cells or inducing apoptosis of T regulatory (T reg) cells.^91^ By contrast, adenosine can activate the adenosine receptors such as A_2b_ receptor and promote tolerogenic DCs.^94^ Thus, blockade of the ectonucleotidases CD39 and CD73 can tilt the balance between immunostimulatory concentrations of eATP and its suppressive metabolic products. Genetic and pharmacological approaches targeting CD39 and CD73 in mice support the potential translation of this axis in cancer immunotherapy.^95–98^

Plasma membrane proteases such as ADAM17/TACE are involved not only in the proteolytic cleavage and generation of oncogenic ligands such as EGFR ligand TGFα, but also in shedding of cell surface molecules including MICA/B and FcγRIIA that can help cancer cells evade NK cells.^99 100^ Proteases such as ADAM17/TACE have complex effect on inflammation as their substrate-derived products can boost inflammation such as through CD154, soluble TNFα and IL-6R, as well as dampen T cell activation such as by cleaving cell adhesion molecules as L-selectin (CD62L). An ADAM17/TACE inhibitory antibody INCB7839 (Aderbasib), in combination with rituximab, is in phase I/II clinical trial as consolidation therapy after autologous hematopoietic cell transplant for patients with diffuse large B cell lymphoma (clinicaltrials.gov identifier: NCT02141451).

Intracellular negative regulators of innate immunity, in addition to plasma membrane inhibitory receptors and ectoenzymes, have come to the forefront in the development of new cancer immunotherapies. Examples of cytoplasmic inhibitory molecules with distinct mechanisms of action abound, from ubiquitin ligase, kinases and phosphatases to metabolic enzymes, to name a few.^101–105^ Following the same paradigm as the inhibitory pathways described above, intracellular negative regulators are engaged as a consequence of immune activation. For example, inflammation induces the expression of indoleamine 2,3-dioxygenase 1 (IDO1) in DCs, a rate limiting enzyme in the metabolism of tryptophan into kynurenine.^105^ Kynurenine and related metabolites are potent suppressors of effector T cells and inducers of T reg cells.^105^ The immunosuppressive function of tryptophan metabolites in preclinical cancer models, the poor prognosis of patients with cancer with high IDO1 activity and the motivation for generating complementary approaches to T cell checkpoint inhibitors propelled the development of multiple IDO1 inhibitors, including indoximod (NewLink Genetics), epacadostat (Incyte) and BMS-986205 (Bristol-Myers Squibb).^106 107^ Unfortunately, despite encouraging preliminary antitumor activity and safety profile of the IDO1 inhibitor Epacadostat in a phase 1 trial, administration of this IDO1 inhibitor together with pembrolizumab did not improve either the progression-free survival or the overall survival in comparison to placebo plus pembrolizumab in patients with unresectable or metastatic melanoma.^108^

Kinases, both receptor tyrosine kinases (RTKs) and intracellular kinases, have also emerged as negative regulators of innate immunity and targeted for ICT. The function of the TYRO3, AXL and MERTK RTKs in the regulation of antitumor immunity is discussed next. Intracellular kinases, such as PI3Kγ, have been found to limit the inflammatory response of macrophages.^103^ Specifically, genetic ablation of PI3Kγ in macrophages led to increased production of inflammatory cytokines such as IL1β and TNFα, but reduced expression of IL-10 or activity of ARG1.^103^ These inflammatory changes correlated with a restoration in CD8+ T cell activation and synergism with ICT in mouse models of cancer.^103^ Kinases are ideal targets for pharmacological intervention with small molecule inhibitors. Indeed, the revolution of targeted therapy in cancer was founded in the development of small molecule kinase inhibitors. A small molecule inhibitor of PI3Kγ (IPI-549, Infinity Pharmaceuticals) given in combination with anti-PD1 and nanoparticle albumin-bound paclitaxel is under investigation in a phase 2 clinical trial in patients with front-line triple-negative breast cancer (clinicaltrials.gov identifier: NCT03961698). Other combinations, such as IPI-549 together with the dual adenosine A_2a_ and A_2b_ receptor inhibitor (AB928, Arcus Biosciences), are also being tested for safety and tolerability (clinicaltrials.gov identifier: NCT03719326).

## Inhibition of checkpoints at the interface of innate and adaptive immunity for improving antitumor responses

The examples described above include tumor cell-derived ligands, tumor microenvironment-derived ligands and autologous mechanisms within T cells and innate immune cells that signal to suppress adaptive and/or innate immunity. Additionally, we described molecules produced by innate immune cells such as IDO that can dampen adaptive immunity. Now, we will discuss how adaptive immunity can also influence innate immunity.

Innate immunity is essential for inducing the adaptive response.^27^ However, once engaged, adaptive immunity has the advantage over innate immunity in terms of the specificity of the response. Antigen specificity of adaptive immunity, in concert with dedicated mechanisms to cull responses against self-antigens, reduces the prospects of self-harm. In contrast, chronic or exaggerated innate immunity can be damaging to the host. Therefore, unsurprisingly, signals derived from cells responsible for adaptive immunity can negatively feedback on innate immune cells to regulate the overall magnitude of the immune response.

V-domain immunoglobulin suppressor of T cell activation (VISTA) is a well-known T cell checkpoint.^109^ CA-170 (Curis), a small molecule targeting PD-L1 and PD-L2, also targets VISTA (clinicaltrials.gov identifier: NCT02812875). Johnson & Johnson developed a fully humanized monoclonal antibody (JNJ-61610588), although the study to determine its safety and tolerability was terminated before completion (clinicaltrials.gov identifier: NCT02671955). VISTA is expressed in myeloid cells as well as in cancer cells and acts on VISTA receptor to suppress the proliferation and cytokine production in CD4^+^ and CD8^+^ T cells.^109^ VISTA is also expressed in CD4^+^, CD8^+^ and Foxp3^+^ T reg cells. While this can function in suppressing T cell activation in an autologous manner, a study using imiquimod-induced murine psoriasis demonstrated that VISTA inhibits the activation of DCs and the production of IL-23 following TLR 7 activation.^110^ The same study also demonstrated that VISTA negatively regulates the activation of IL-17-producting γδ T cells and Th17 cells.^110^ Therefore, it is conceivable that at least some aspects of VISTA blockade in boosting antitumor immunity involve releasing the brakes on innate immunity.

A well-known mechanism that functions at the interface of innate and adaptive immunity is the PROS1-TYRO3/AXL/MERTK RTK signaling axis.^102^ Once DCs activate antigen-specific T cells, these activated T cells express PROS1,^111 112^ which is a ligand for the RTKs TYRO3, AXL and MERTK (collectively termed TAM RTKs). There are, in fact, two ligands for the TAM RTKs: GAS6 and PROS1. GAS6 has the highest affinity for AXL, but can also activate TYRO3 and MERTK.^102^ PROS1 can activate MERTK and TYRO3, but not AXL.^102^ PROS1, but not GAS6, was found to be expressed by activated, and not by resting, murine T cells.^111^ An additional feature of the TAM ligands is that they contain a vitamin K-dependent carboxylation/gamma-carboxyglutamic acid (Gla)-domain. This domain binds phosphatidylserine (PtdSer) when exposed on the outer leaflet of the cell surface plasma membrane. Activated, but not resting T cells transiently expose PtdSer.^111^ Therefore, T cell-derived PROS1 may be displayed on its surface through PtdSer binding. This PtdSer-bound PROS1, in turn, binds MERTK and/or TYRO3 in DCs. The net result is a dampening of DC activation.^111^ On one hand, PROS1-MERTK signaling appears to be more relevant in a type I immune response setting where its effect on DCs were to lower levels of MHC II expression, decrease expression of costimulatory molecules CD80 and CD86 and reduce the production of cytokines such as IL6 and TNFα.^111^ On the other hand, PROS1-TYRO3 signaling was observed in PDL-2^+^ DCs associated with a type II immune response and resulted in the dampening of the cytokines IL-4, IL-5 and IL-13, and the chemokines CCL17, CCL22 and CCL5.^113^ Disabling the PROS1-MERTK axis enhances antitumor immunity. *Mertk^−^* ^/−^ mice have been shown to be more resistant to tumor growth in various murine tumor models.^114 115^ Whether this is a result of disabling MERTK function specifically in DCs resulting in their enhanced activation or if the loss of MERTK-dependent phagocytic function of macrophages (please see below) also contribute to anti-immunity remains unknown. Interestingly, the genetic ablation of MERTK promotes proinflammatory macrophage polarization.^116^ Similar results were observed in tumor-infiltrating leukocytes. In syngeneic mouse models of breast cancer, melanoma and colon cancer, tumor-associated *Mertk* ^−/−^ CD11b^+^ cells produced more IL-1β, IL-6 and IL-12p40 than their *Mertk* ^+/+^counterparts, suggesting that these macrophages were more proinflammatory.^114^ Tumors grew slower and were less metastatic in *Mertk* ^−/−^mice, in comparison to wild-type control mice.^114^ This protective effect was shown to be dependent on *Mertk* ^−/−^ bone marrow.^114^ Although macrophage-specific *Mertk* deletion was not used in this study, the results are consistent with the notion that the proinflammatory macrophage phenotype enabled an improved CD8^+^ T cell response, as antibody-mediated depletion of CD8^+^ T cells abolished the acquired antitumor immunity in *Mertk* ^−/−^mice.^114^ Consistent with these results, tumor cells upregulate GAS6 and PROS1 expression and PROS1, but not GAS6, inhibited LPS+IFNγ-driven macrophage production of IL-1 and IL-6.^117^ Coculture of B16F10 mouse melanoma cells with LPS+IFNγ-polarized macrophages from wild-type and *Axl* ^−/−^ mice, but not *Mertk* ^−/−^ or *Tyro3* ^−/−^ mice, resulted in inhibition of IL-1 and IL-6 expression.^117^ Thus, inhibition of TAM signaling improves the antitumor function of DCs, as well as macrophages. While enhanced type II immunity has been shown to drive antitumor immune responses in some settings,^118 119^ it remains to the be tested if inhibiting the PROS1-TYRO3 axis at the DC:T cell interface also can boost antitumor immunity.

A number of TAM receptor inhibitors are currently in clinical trials and preclinical development. These include BGB324/bemcentinib (BergenBio) in NSCLC (clinicaltrials.gov identifiers: NCT02424617 and NCT02922777), in pancreatic cancer (clinicaltrials.gov identifier: NCT03649321), in AML and myelodysplastic syndromes (clinicaltrials.gov identifier: NCT02488408) and in glioblastoma (clinicaltrials.gov identifier: NCT03965494). Another small molecule inhibitor, TP-0903 (Tolero Pharmaceuticals), is also in multiple clinical trials (clinicaltrials.gov identifiers: NCT02729298 and NCT03572634). MGCD265/Glesatinib (Mirati Therapeutics) is another small molecule tyrosine kinase inhibitor that targets MET and TAM receptors. It is being evaluated in NSCLC (clinicaltrials.gov identifier: NCT02544633). Safety, tolerability and efficacy studies with a dual AXL-MERTK inhibitor, ONO-7475 (ONO Pharmaceutical Company), are underway in AML (clinicaltrials.gov identifier: NCT03176277). MRX-2843 (Meryx) is a MERTK-FLT3 inhibitor undergoing similar trials in advanced solid tumors (clinicaltrials.gov identifier: NCT03510104). AVB-S6-500 (Aravive Biologics), an AXL Fc-fusion protein that binds GAS6, gained FDA fast track designation as potential treatment of platinum-resistant recurrent ovarian cancer following a phase 1/b study (clinicaltrials.gov identifier: NCT03639246).

## Neutralizing myeloid-derived suppressor cells (MDSCs) to enhance antitumor immunity

Absent in healthy individuals at baseline, MDSCs are myeloid cells resembling neutrophils and monocytes that are enriched with chronic inflammation. MDSCs are potent suppressors of the immune response.^120 121^ Cancer-associated Gr1^+^ CD11b^+^ are a heterogenous population of cells composed of polymorphonuclear (CD11b^+^ Ly6G^+^ Ly6C^lo^ PMN-MDSC) and monocytic (CD11b^+^ Ly6G^−^ Ly6C^hi^ M-MDSC) subsets that are correlated with poor overall and progression-free survival of the patients.^122^ MDSCs can mediate immune suppression via both antigen-specific T cell suppression such as by ROS production and nitration of T cell-receptors, as well as non-specific mechanisms to suppress T cell functions including the production of ARG1, iNOS, TGFβ, IL-10, COX2, IDO and other factors.^120 121 123 124^ Not surprisingly, MDSC levels have been associated with patient response to anti-CTLA4 and anti-PD-1 therapy.^125–128^ MDSC numbers can be reduced by gemcitabine, 5-fluorouracil, PDE-5 inhibitor tadalafil, class 1 HDAC inhibitor entinostat, all-trans retinoic acid, and by targeting the TRAIL receptor, CXCR2, TNFα/TNFR1, CSF1 and IL-18.^127 129–141^ Additionally, STAT3 inhibition can potentiate MDSC differentiation into DCs.^142 143^

Interestingly, both PMN-MDSCs and M-MDSCs upregulate AXL, MERTK and TYRO3, as well as their ligands GAS6 and PROS1 in a subcutaneous mouse model of melanoma.^144^ Consistent with this finding, the frequency of MERTK^+^ and TYRO3^+^ PMN-MDSCs and M-MDSCs was increased in patients with metastatic melanoma.^144^ Genetic ablation of *Axl*, *Mertk* or *Tyro3* individually in mice led to reduced ARG1, TGFβ and ROS production in both types of MDSCs and iNOS and IDO is M-MDSCs.^144^ iNOS was also reduced in PMN-MDSCs in *Axl* ^−/−^ and *Tyro3* ^−/^^−^ mice and IDO in in PMN-MDSCs in *Axl* ^−/^^−^ and *Mertk* ^−/^^−^ mice. In in vitro assays, the loss of TAM RTKs and their ligands reversed MDSC-induced suppression of T cell proliferation and also improved differentiation of MDSCs into DCs and macrophages.^144^ A TAM RTK inhibitor UNC4241, by itself and in combination with anti-PD-1, reduced tumor volume, but only the combination increased survival in the mouse model of melanoma.^144^

## Molecules that license T cell entry into tumor microenvironment as targets to improve antitumor immunity

Innate immune checkpoints are not only important for initiating a more robust antitumor immune response, molecules expressed in endothelial or stromal cells that license T cell infiltration into the tumor parenchyma may also function as checkpoints. T cells extravasate and home to inflamed tissue by at least three, distinct steps: T cell rolling on endothelium mediated by selectin interactions, activation of integrins through chemokine signaling and integrin-dependent transmigration. Therefore, activation of molecules expressed by endothelial cells that allow and/or instruct T cell extravasation and infiltration, or inhibition of those that prevent these processes, may also effectively function in boosting ICT. For example, upregulation of E-selectin or P-selectin in tumor-associated endothelia might allow improved homing of T cells expressing E-selectin and P-selectin ligands. Similarly, increased CXCL9 and CXCL10 produced by tumor endothelia may enhance the recruitment of T cells via CXCR3 and activate the leukocyte integrins LFA-1 and VLA-4 for binding ICAM-1 and VCAM-1.^145^ In contrast, CD155 or galectin 9 expressed in endothelial cells inhibit effector T cell activation via TIGIT and TIM3, respectively.^7 146–150^ Therefore, blockade of such inhibitory molecules may enhance ICT.

Not only endothelia-derived molecules, but also molecules produced by stromal cells can license or prevent the presence of T cells at the tumor site. A TGFβ signature in cancer-associated fibroblasts was associated with exclusion of CD8^+^ T cells from the tumor parenchyma in patients with anti-PD-L1 (atezolizumab) non-responsive metastatic urothelial cancer.^26^ Instead, the fibroblast-rich and collagen-rich peritumoral stroma was enriched in CD8^+^ T cells in these patients, suggesting a distinct licensing event for T cells to infiltrate the tumor parenchyma.^26^ Depletion of tumor-associated mesenchymal cells expressing fibroblast activation protein-α (FAP) resulted in suppression of immunogenic Lewis Lung Carcinoma (LLC) tumor growth, but not of non-immunogenic tumors.^151^ Although FAP^+^ mesenchymal stromal cells did not alter the proportions of CD8^+^ and CD4^+^ T cell or of Foxp3^+^ T reg cells, they inhibited the production of TNFα and IFNγ in the tumor microenvironment.^151^ Similar results were observed with a pancreatic ductal adenocarcinoma mouse model.^151^ A chemokine mediating the immunosuppressive effects of FAP^+^ mesenchymal stromal cells in this autochthonous model was CXCL12.^152^ A CXCL12 receptor inhibitor, AMD3100, synergized with anti-PD-L1 for an effective antitumor immune response.^152^

Finally, the tumor-associated vasculature may be the target of efforts to improve cancer immunotherapy. The physical nature of tumor angiogenesis, characterized by lack of pericyte coverage of vessels, tortuous vessel path and leakiness, may exclude T cells from approaching the tumor.^153^ The genetic deletion of RGS5 in mice not only restores pericyte maturation and results in vessel normalization, but also improves CD8^+^ and CD4^+^ T cell trafficking after adoptive transfer to tumor parenchyma in RIP1-Tag5 mouse tumor model.^154^ This correlated with improved survival of tumor-bearing mice.^154^

TAM RTK signaling may also function in endothelial cells and in the tumor microenvironment. GAS6 has been demonstrated to drive proliferation and prevent apoptosis in vascular smooth muscle cells.^155–157^ Another study showed that migration of human umbilical vein endothelial cells was significantly reduced when the expression of *Axl* or its agonist *Gas6* were silenced.^158^ *Axl*-deficient mice have impaired blood vessel formation and function, indicating the importance of AXL in angiogenesis.^159^ However, other studies demonstrated a somewhat contradictory role of AXL in angiogenesis. For example, Gallicchio *et al* described an AXL-dependent inhibitory role of GAS6 in VEGFA-VEGFR2-dependent angiogenesis.^160^ The endothelial/vasculature functions of GAS6 in the context of tumors have not been characterized. GAS6, as well as the TAM RTKs, also have direct effects on promoting tumor growth.^102^ Tumor-infiltrating leukocytes upregulate GAS6 and support tumor growth.^161^ Taken together with its role in the interface of innate and adaptive immunity, the neutralization of TAM ligands or the inhibition of TAM RTK signaling might mediate tumor killing via multiple mechanisms.

## Sensing and processing dead cells for antitumor immunity

A physiological immune response not only fights off the foreign invader while restraining itself so as not to excessively injure the host tissue through exaggerated inflammation, but also resolves and allows tissue repair. We posit that cell death can function as a novel checkpoint where the immune response transitions from being on a warpath to adopting a role supporting tissue repair and restitution. The later might abet tumor progression. Cancer has been described, originally by Harold Dvorak in 1986, as ‘wounds that do not heal’.^162^ In fact, the historical paper of Kerr *et al* published in 1972 that coined the term ‘apoptosis’ reported widespread apoptotic cell death in malignant neoplasms including rectal adenocarcinoma and squamous cell carcinoma of the human cervix uteri.^163^ Therefore, the abnormal and perhaps continuous presence of cell death, or the response to it, might force a premature transition of the immune response to its tissue repair mode and prevent a consistent proinflammatory environment favoring the generation of an antitumor T cell immune response. For example, we have previously shown that macrophages transition to a tissue-repair phenotype in the presence of apoptotic cells and IL-4.^116^ This is achieved through the TAM RTK signaling that is known to mediate phagocytosis of apoptotic cells—termed efferocytosis—by macrophages. The ligands for TAM RTK—GAS6 and PROS1—contain Gla domains, which when γ-carboxylated in a vitamin K-dependent manner, bind PtdSer in apoptotic cells, effectively bridging the dying cells to TAM RTKs on macrophages.^102^ Therefore, blocking apoptotic cell death recognition by TAM RTKs may function as a novel mechanism of checkpoint blockade to boost the antitumor T cell responses.

The beneficial effects of blocking apoptotic cell death sensing is likely to extend beyond TAM RTK function. PtdSer is exposed on the outer leaflet of dying cells and serves as a ligand for a number of receptors including TIM-3 and TIM-4.^164^ TIM-4 is expressed in cancer tissue, including in colorectal cancers and NSCLC.^165 166^ While TIM-4 is known to be expressed in tumor-associated macrophages and DCs in B16F10 mouse model of melanoma,^167 168^ and in fact, is known to signal through MERTK,^169^ only tumor cell-intrinsic functions were described in the colorectal cancer and the lung cancer studies.^165 166^ By contrast, an immunological mechanism was described in the B16F10 mouse model of melanoma.^167 168^ The upregulation of TIM-4 on tumor-associated myeloid cells was reported to be induced by the release of danger-associated molecular patterns (DAMPs) from chemotherapy-damaged tumor cells.^168^ TIM-4 activated autophagy-mediated degradation of tumor material in the tumor-associated myeloid cells.^168^ This resulted in reduced antigen presentation and impaired cytotoxic T cell responses against the tumors, including reduced IFNγ^+^ T cells.^168^ Blockade of TIM-4 augmented the benefits of chemotherapy and increased tumor-specific cytotoxic T cell response.^168^ The same group also demonstrated that blockade of TIM-3 and TIM-4 using antagonistic antibodies enhanced the response of B16 melanoma to a vaccination protocol with irradiated B16 cells expressing FLT3L.^167^ It remains to be determined if ablation or inhibition of efferocytosis by TIM-4 also alters macrophage polarization and thereby contributes to the antitumor response. Another PtdSer receptor BAI1 has been shown to drive a proinflammatory anti-gram-negative bacterial macrophage response against *Salmonella enterica* serotype typhimurium,^170^ as well as an antiviral macrophage response against oncolytic herpes simplex virus.^171^ Whether BAI1-dependent efferocytosis has any role in tumor progression remains unknown. While macrophages are considered the major undertakers and therefore the effectors of PtdSer-directed interventions, it was described that treatment of mouse melanoma models by a combination of anti-PtdSer antibody and anti-PD-1 resulted in reduction of MDSCs and enhancement of antitumor immune response.^172^ In conclusion, blocking the sensing of apoptotic cell death may drive an increasingly proinflammatory, antitumor immune response.

In contrast to apoptotic cell death, inducing non-apoptotic programmed cell death such as ferroptosis or necroptosis can synergize with ICT. An inducer of ferroptosis, cyst(e)inase, synergized with anti-PD-L1 in reducing tumor growth in mice bearing ID8 ovarian tumor cells.^173^ PD-L1 blockade alone also induces ferroptotic cell death and inhibition of this form of programmed cell death attenuated the effects of PD-L1, and PD-1 and CTLA-4 blockade.^173^ Interestingly, Jurkat cells undergoing ferroptosis expressed comparatively reduced levels of PtdSer relative to apoptotic cells, and were engulfed less efficiently by human peripheral blood monocyte-derived macrophages in comparison to apoptotic and necroptotic cells.^174^ Necroptotic cell death of tumor cells in LL2 lung carcinoma or B16F10 melanoma mouse models or in the tumor microenvironment in LL2 lung carcinoma, B16F10 melanoma and E.G7 thymoma mouse models also reduced tumor growth.^175^ Necroptosis in the tumor microenvironment created an environment for enhanced antigen uptake by tumor-associated antigen presenting cells and, tumor control required Batf3^+^ DCs and CD8^+^ T cells.^175^ Additionally, this necroptosis synergized with anti-PD-1 and generated long-term memory.^175^ Prostaglandins such as PGE2 is released on cell death and functions as an inhibitory DAMP.^176^ PGE2 in the tumor microenvironment reduced NK cell survival and their function in recruiting Batf3^+^ DCs through CCL5 and XCL1.^43^ The reduction in NK cell number and Batf3^+^ DCs function resulted in cancer immune evasion.^43^ Thus, certain modalities of cell death, the mechanisms of clearance of the corpses or factors associated with cell death may dampen ICT response while others favor its beneficial effects. Importantly, it is not only tumor cells that die during therapies including ICT, but also stromal and immune cells. Therefore, even the death of immune and/or stromal cells by a specific modality provides a positive feedback for antitumor immunity.^175^

Sensing and uptake of cellular corpses is followed by degradation of the cargo and its metabolic processing. The degradation of the engulfed cargo relies on phagosome maturation. This is a complex process that in some settings involves components of the autophagy machinery and is known as LC3-mediated phagocytosis or LAP.^177^ Similar to the results from the activation of the efferocytosis receptors TAM and TIM-4 described above, engagement of LAP suppresses the inflammatory response.^178 179^ Consistently, genetic ablation of LAP components in myeloid cells led to increase resistance to tumor growth in multiple models including B16F10 melanoma, LLC, MC38 adenocarcinoma and Kirsten rat sarcoma oncogen (KRAS)-driven lung cancer.^178^ The antitumor response in mice deficient in LAP was characterized by an increase in STING-dependent production of type I IFNs and cytotoxicity of CD4^+^ and CD8^+^ tumor-infiltrating lymphocytes.^178^ It should be noted that LAP-deficient phagocytes are still able to engulf dying cell. It is conceivable that the processing of the engulfed cargo is aberrant in LAP-deficient cells and leads to a STING-dependent anti-tumor immunity.

## Concluding remarks

ICT need not rely solely on the blockade of T cell checkpoints. At least in theory, all negative regulatory nodes that restrain the immune response can be targeted for improving ICT. Therefore, there is still a lot of real estate to explore in this area. It remains to be determined whether innate immune checkpoint blockade would be effective on its own as a therapeutic modality, or whether its beneficial effects may be counteracted by T cell exhaustion. In a scenario wherein T cell exhaustion limits innate immune checkpoint blockades, these novel immunotherapy modalities could be combined with T cell checkpoint inhibitors. Another area of concern is immune-related adverse events. Negative regulators or checkpoints likely evolved to limit host damage due to exaggerated immune response. Even approved T cell checkpoint inhibitors can be associated with immune-related adverse events.^180^ Such events may represent a significant hurdle for strategies that let off the brakes of innate immunity. Perhaps transient or limited release of these brakes can empower the immune system to fight cancer while avoiding severe immune-related adverse events. Nevertheless, the future of ICT appears poised for a rapid expansion.
