# Supplementary material for: Lifting the innate immune barriers to antitumor immunity
Source: J Immunother Cancer. 2020 Apr 8;8(1):e000695. doi: 10.1136/jitc-2020-000695 (PMC7254113; doi:10.1136/jitc-2020-000695)
Supplement: Supplementary data [file jitc-2020-000695supp001.pdf]

Table 1: Active, not yet recruiting, recruiting, enrolling by invitation and completed NCI-approved clinical trials related to indicated targets as listed in clinicaltrials.gov by February, 2020. Target molecules, immune cell expression based on Immgen Gene Skyline database, therapeutic agents and clinical trial number are listed. Note that Clinical studies of FDA-approved drugs, such as bosutinib, sunitinib and cabozantinib (targeting receptor tyrosine kinases including the TAM receptor tyrosine kinase inhibitors) are not included in the table. Abbreviations: LAG3, lymphocyte activation gene 3; TIM3, T cell membrane protein 3; VISTA, V-domain immunoglobulin suppressor of T-cell activation; KIR, killer cell immunoglobulin-like receptor; TLR, Toll-like receptor; FLT3, fms-like tyrosine kinase 3; DC, dendritic cells; NK, natural killer; NKT, natural killer T cells; NCT, national clinical trial; CAR NK,chimeric antigen receptor–directed natural killer cells.
